# Universal high-sensitivity CAR T-cell monitoring by targeting linker sequences

**DOI:** 10.3389/fimmu.2026.1787951

**Published:** 2026-03-25

**Authors:** Nora Rebecca Schwingen, Lina Meretuk, Michael Aigner, Sascha Kretschmann, Julia Katharina Scholz, Franziska Gsottberger, Dennis Christoph Harrer, Janin Dingfelder, Gloria Lutzny-Geier, Soraya Kharboutli, Andreas Mackensen, Fabian Müller, Simon Völkl

**Affiliations:** 1 Department of Internal Medicine 5, Hematology and Oncology, Friedrich-Alexander-Universität Erlangen-Nürnberg and Universitätsklinikum Erlangen, Erlangen, Germany; 2 Bavarian Cancer Research Center (BZKF), Erlangen, Germany; 3 Deutsches Zentrum Immuntherapie (DZI), Friedrich-Alexander-Universität Erlangen-Nürnberg and Universitätsklinikum Erlangen, Erlangen, Germany; 4 Department of Internal Medicine III, Hematology and Internal Oncology, University Hospital Regensburg, Regensburg, Germany

**Keywords:** CAR (chimeric antigen receptor) T cells, CAR construct, CAR T-cell therapy, diagnostic test, immune monitoring, linker

## Abstract

**Introduction:**

Chimeric antigen receptor (CAR) T-cell therapy has become a standard-of-care in oncology, yet standardized monitoring of circulating CAR T cells remains a major challenge due to diverse CAR constructs and limited availability of detection reagents. The antibody domain of the CAR commonly consists of the heavy and light chain connected through a linker, typically either 4x glycine and 1x serine (G4S) or a defined amino acid sequence (Whitlow/218). Here, we evaluated the novel monoclonal antibodies (mAbs) targeting the linker sequence as a universal tool for CAR detection.

**Methods:**

Using flow cytometry, we compared anti-linker mAbs with conventional reagents, including anti-idiotype CD19.FMC63 mAb, CD19 and BCMA antigen-based detection reagents (Ag), and anti-F(ab′)_2_ mAb. Analyses were performed on commercial CD19- and BCMA-directed CAR T-cell products and an investigational Claudin-6 (CLDN6) CAR T-cell product. Performance was further assessed across diverse experimental platforms for clinical monitoring and in a murine model to evaluate sensitivity and translational relevance.

**Results:**

Linker-mAbs detected all tested CAR constructs with high specificity and sensitivity, matching target-specific binding with conventional Ag reagents. Anti-linker mAbs demonstrated minimal background and low limits of quantification both comparable to Ag reagents. Longitudinal monitoring in lymphoma and myeloma patients revealed consistent CAR T-cell kinetics between anti-linker mAbs and Ag reagents. High performance of linker-based CAR-detection was demonstrated in high-dimensional, multi-parameter flow cytometry, in immunofluorescence imaging and in a murine model of anti-CD19 CAR T cells.

**Conclusion:**

These findings establish anti-Whitlow/218 and anti-G4S mAbs as sensitive, specific, and universal reagents for CAR detection across multiple targets, constructs, and species, providing a standardized platform for harmonization of CAR T-cell monitoring in preclinical, clinical trial, and diagnostic settings.

## Introduction

Over the past decade, chimeric antigen receptor (CAR) T-cell therapy has emerged as a transformative approach in oncology, achieving remarkable clinical success in patients with B-cell leukemia, lymphoma, and multiple myeloma (MM) (1, 2). More recently, their application has expanded beyond hematologic malignancies to include solid tumors and increasingly autoimmune disorders (3–7). Most clinically approved CAR T-cell products are based on second-generation CARs which are composed of four main parts: an extracellular antigen-recognition domain, typically a single-chain variable fragment (scFv), a hinge and transmembrane region, and an intracellular signaling module combining a costimulatory domain, commonly CD28 or 4-1BB, with the CD3ζ activation domain (8, 9). The scFv is constructed from the variable heavy (VH) and light (VL) chain of an antibody, which are typically linked through either a repeat of four glycines followed by a serine (G4S) or by a Whitlow linker (10–12). Currently, six CAR T-cell products are commercially available in Germany: Kymriah (tisagenlecleucel, tisa-cel), Yescarta (axicabtagene ciloleucel, axi-cel), Breyanzi (lisocabtagene maraleucel, liso-cel), and Tecartus (brexucabtagene autoleucel, brexu-cel) target the CD19 antigen, whereas Abecma (idecabtagene vicleucel, ide-cel) and Carvykti (ciltacabtagene autoleucel, cilta-cel) target the B-cell maturation antigen (BCMA) (13–18). In addition, the Claudin-6 (CLDN6)-directed CAR T-cell product BNT211 is currently under clinical evaluation (3, 19). As the number of approved and investigational CAR T-cell therapies grow and the pharmacodynamics of CAR T cells are associated with treatment response and toxicities, close and where possible real-time monitoring of circulating CAR T cells has become a key component of both clinical management and translational research (5, 20, 21). Subsequently, several detection approaches have been developed, including PCR-based and flow cytometry-based assays. Flow-based methods utilize anti-idiotype monoclonal antibodies (mAbs), antigen-based detection reagents (Ag), and polyclonal anti-immunoglobulin G (IgG) antibodies recognizing F(ab′)_2_ fragments (22–25). Anti-idiotype mAbs, such as the anti-CD19.FMC63 mAb, targeting the murine scFv sequence used in most CD19-directed CARs, offer high specificity and sensitivity (26, 27). Ag reagents, using recombinant human (rh) proteins that bind the CAR recognition domain, enable precise detection but are restricted to the corresponding antigen-binding CAR (24, 25). Conversely, polyclonal anti-IgG antibodies provide broader applicability by recognizing multiple epitopes, which frequently comes at the cost of reduced binding specificity due to higher non-specific background signal (24). These detection strategies differ substantially in binding characteristics and sensitivity, hindering comparability across studies, CAR constructs, and clinical centers (24, 28). Furthermore, next-generation CAR T-cell therapies with novel scFv designs or species-specific backbones, as used in emerging murine models, often lack compatible detection reagents, complicating both preclinical and clinical analyses (29–32). Recently, mAbs targeting the linker regions between the VH and VL chains of standard scFv-based CAR constructs have been proposed as universal CAR detection tools. Since most CARs employ either the Whitlow/218 linker (GSTSGSGKPGSGEGSTKG) or the Gly_4_Ser (G4S) linker (GGGGSGGGGSGGGGGS), mAbs directed against these sequences (anti-Whitlow/218 and anti-G4S) may enable target-independent CAR detection (10–12, 22, 33).

Aiming to harmonize and standardize the detection and monitoring of increasingly diverse CAR T-cell constructs, we systematically evaluated the specificity and sensitivity of the anti-Whitlow/218 and anti-G4S mAbs across various CAR T-cell products, including commercial, investigational, and murine CARs, compared their performance against commonly used anti-idiotype and antigen-based detection reagents, and evaluated their use beyond flow cytometry also in immunofluorescence.

## Methods

### Patient samples and preparation

Lymphocytes were obtained from healthy donors (HD) and patients receiving CAR T-cell therapy, including non-Hodgkin lymphoma, multiple myeloma, autoimmune and solid tumors 4–169 days post-infusion. Detailed patient characteristics and sampling time points are summarized in **Supplementary Table 1**. Peripheral blood mononuclear cells (PBMCs) were isolated from EDTA-anticoagulated blood using Pancoll density gradient centrifugation (PAN-Biotech), washed twice with PBS (ThermoFisher), and stored in 90%FCS/10% DMSO in liquid nitrogen until use. Commercial CAR T-cell products analyzed prior to infusion included: tisagenlecleucel (Kymriah^®^), axicabtagene ciloleucel (Yescarta^®^), ciltacabtagene autoleucel (Carvykti^®^), and idecabtagene vicleucel (Abecma^®^). Additionally, samples were taken from patients treated as part of the BNT211–01 NCT04503278 clinical trial (BNT211 – CLDN6 CAR T-cell therapy) and the investigational medicinal product MB-CART19.1 (in-house manufactured). Detailed construct characteristics are summarized in **Supplementary Table 2**. All samples of patient and of healthy donors were collected after written informed consent, with approval by the local ethical committee (#19-336_1-B, 24-487-Bp, 59_17 Bc), and were used in accordance with the Declaration of Helsinki.

### Generation of murine CAR T-cells

Splenocytes were harvested from C57BL/6 mice and T cells were isolated by negative selection using pan T cell isolation kit II (Miltenyi Biotec, Germany) according to the manufacturer’s protocol. Purified T cells were activated on plates pre-coated with 2.5 µg/mL anti-mouse CD3ϵ (clone 145-2C11, BioXCell) in the presence of 1 µg/mL soluble anti-mouse CD28 (clone 37.51, BioXCell) and 80 U/mL recombinant human IL-2 (Peprotech, Cat. 200-02). Activated T cells were transduced with the MSGV1-1D3-28Z.1-3mut retroviral vector (Addgene, Cat. 107227) (31) encoding an anti-murine CD19-specific CAR. Retrovirus was immobilized on RetroNectin-coated plates (Takara Bio, Cat. T100B) and spinoculation was performed at 800 x g for 30 min at 32 °C. After the centrifugation, T cells were incubated for 48 h, all cells harvested from the bottom of the well, and stained with anti-mouse antibodies as listed in **Supplementary Table 3**. CAR expression was detected using a polyclonal goat anti-rat IgG, F(ab′)_2_ fragment as previously described (31) and compared with the anti-G4S mAb.

### Immunofluorescence staining of co-cultured cells

HS-5 stromal cells were seeded into 8-well chamber slides (IBIDI, Germany), cultured until confluence and co-cultured with chronic lymphocytic leukemia (CLL) cells for 3 days. CD19-directed CAR T cells, manufactured as previously described (34), were added at an effector-to-target (E:T) ratio of 1:1, and incubated for 18 hours. Co-cultures were washed twice with PBS, fixed with 4% paraformaldehyde for 10 minutes and permeabilized with 0.1% Triton-X100 for 15 minutes at room temperature (RT). Cells were incubated in blocking buffer (1% BSA, 0.2% Triton X-100, 5% FCS in PBS) for 2 hours and stained overnight at 4 °C with an anti-CD19 mAb (1:100) and the anti-Whitlow/218 linker (1:50). After washing, cells were incubated with anti-rat Alexa Fluor 594 for 2 hours, followed by Flash Phalloidin™ Green 488 staining for 20 min at RT in the dark. Used antibodies are listed in **Supplementary Table 3**. Co-cultures were washed, mounted with DAPI-containing mounting medium (IBIDI, Germany), dried overnight, and stored at 4 °C. Confocal laser imaging was performed on a Leica Stellaris 8 (Wetzlar, Germany) using Fiji with the 3Dscript plugin for 3D visualization of cell layers (35, 36).

### Flow cytometric staining and detection of CAR constructs

For flow cytometric detection of CAR^+^ T cells, PBMCs were thawed, washed twice and stained with the respective detection reagent. Detection antibodies were added at different times depending on their binding characteristics, anti-F(ab′)_2_, anti-BCMA Ag, and anti-CD19 Ag were incubated for 10 min at RT and washed twice prior to surface staining to allow optimal binding to CAR epitopes, whereas anti-idiotype CD19.FMC63 mAb, anti-linker mAbs or anti-Biotin were added together with surface antibodies incubated for 10 min at RT and washed once, to prevent steric hindrance and preserve epitope accessibility. Brilliant Stain Buffer Plus (BD Bioscience, Germany) was added for surface staining of the high-dimensional 17-color panel. For intracellular staining, cells were treated with Cytofix/Cytoperm for 20 min at 4 °C in the dark and washed twice with Wash/Perm buffer (both BD Bioscience). After incubation with intracellular antibodies for 30 min at 4 °C in the dark, cells were washed twice with Wash/Perm buffer and fixated with Fluorofix buffer (Biolegend, USA). All antibodies used for flow cytometric analyses are indicated in **Supplementary Table 3**. Flow cytometry data were acquired on an LSR Fortessa or FACSCanto (BD Biosciences, Germany).

### Statistical analysis

Flow cytometric data was analyzed using the software Kaluza Analysis 2.1 (Beckman Coulter Life Sciences, USA) or FlowJoTM software version 10.9 (BD Biosciences, Germany). Statistical and graphical analysis was performed using GraphPad Prism v.10.2 (GraphPad). Statistical analysis was determined by one-way ANOVA and accounted for multiple testing using Tukey. If not stated otherwise data are presented as mean ± standard deviation (SD). Comparability of CAR constructs was calculated by Spearman’s rank correlation with a 95% confidence interval.

### High-dimensional flow cytometry data analysis

Flow cytometry data were analyzed using the web-based platform OMIQ (https://www.omiq.ai/). In brief, compensated FCS-files were uploaded and fluorescence parameters were scaled using arcsinh transformation. The time parameter was gated to control for instrument stability, followed by singlet gating (FSC-H vs. FSC-A) and identification of viable T cells (7-AAD^-^CD3^+^). T cells were then downsampled to 75,000 cells per patient in automatic mode followed by uniform manifold approximation and projection (UMAP) dimensionality reduction (settings: 15 neighbors, minimum distance 0.4, Euclidean metric, and 200 epochs). The optimal number of clusters was determined by FlowSOM elbow metaclustering, followed by FlowSOM consensus metaclustering to define phenotypically distinct cell populations. Cluster characteristics were evaluated based on individual marker expression, visualized in the two-dimensional UMAP space and using unsupervised clustered heatmaps. All fluorescence channels, except for 7AAD, were selected as features for UMAP dimensionality reduction, FlowSOM clustering, and heatmap clustering.

### Calculation of sensitivity and specificity of CAR constructs

The stain index (SI) was defined by the ratio of the separation of the stained positive and negative population. For each CAR construct the SI was calculated using the formula (37):


$$\frac{\left(MFI\;CAR^{+})-(MFI\;CAR^{-}\right)}{2*SD\;MFI\;CAR^{-}}$$

To assess the background staining of each CAR construct, the limit of blank (LOB) was determined as the mean of blank specimens + 1.645*SD_Blank_ (26, 38, 39). The limit of detection (LOD) was defined by the lowest concentration reliably distinguished from the LOB as LOB + 1.65*SD_low concentration sample_. The lowest concentration was chosen as a value just above the LOB in which the coefficient of variation between each measurement was less than 30-35% (38, 39). To test the sensitivity of CAR detection reagents, cells from idecabtagene vicleucel (Abecma^®^), BNT211 (anti-CLDN6), tisagenlecleucel (Kymriah^®^) products were used. CAR^+^ T-cell products were serially diluted (100% CAR T-cells dilution, defined as the undiluted final product containing CAR-expressing T cells, to 0.01% CAR T-cell dilution) with PBMCs, obtained from HDs (38). The lower limit of quantification (LLOQ) was defined as the lowest concentration for the detection of specific expression. The value for the LLOQ was determined from the last dilution of CAR^+^ T-cells above the LOB and the LOD (39).

## Results

### Anti-linker mAbs enable universal detection of scFv-based CAR constructs

**Figure 1:**
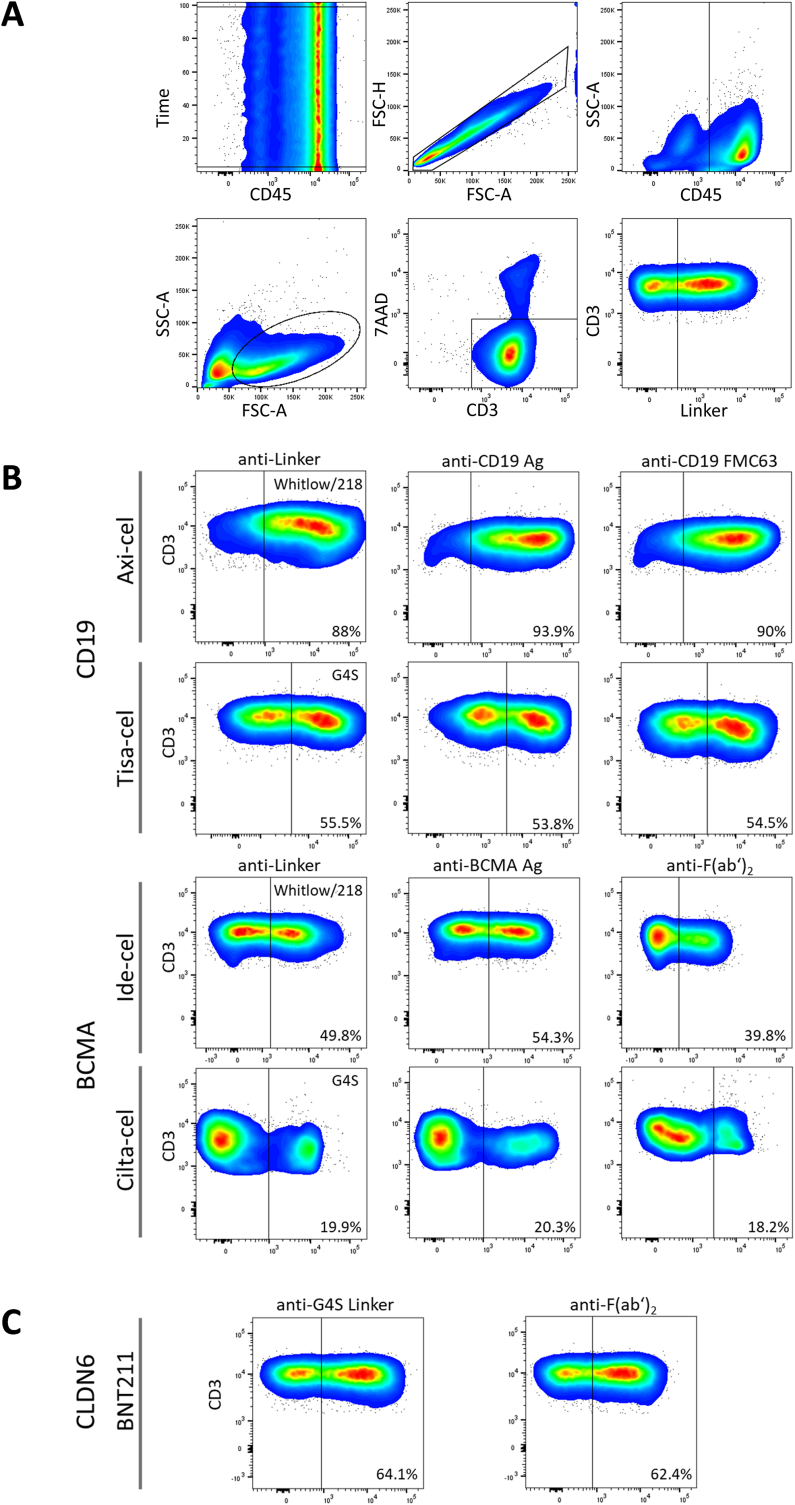
Linker-specific detection of various commercial and investigational CAR T-cell products. **(A)** Gating strategy for the identification of CAR T cells by flow cytometry, shown using representative density plots. CD45 vs. time was gated to control for instrument stability, followed by singlet gating (FSC-H vs. FSC-A) and identification of leukocytes (SSC-A vs. CD45) and lymphocytes (SSC-A vs. FSC-A). Viable T cells were defined as 7-AAD^-^CD3^+^. CAR T cells were subsequently identified as CD3^+^ anti-CAR reagent^+^ double-positive cells. **(B, C)** Representative density plots of CAR T-cell detection of commercial CD19-targeting CAR T-cell products (axi-cel, tisa-cel) and BCMA-targeting CAR T-cell products (ide-cel, cilta-cel) **(B)** as well as in the investigational CLDN6-targeting CAR T-cell product BNT211 **(C)**. CAR expression was detected using monoclonal antibodies against the linker (anti-G4S linker or anti-Whitlow/218 linker, as indicated), an antigen-based anti-CD19 or anti-BCMA reagent (Ag), or anti-idiotype CD19.FMC63, and anti-F(ab′)_2_ mAbs. Density plots depict representative intensity and rate of CAR^+^ T cell staining within gated CD3^+^ T-cell populations.

As most CAR constructs incorporate a G4S or Whitlow/218 linker sequence we evaluated an anti-linker CAR detection approach by comparing flow cytometric analyses using anti-Whitlow/218 and anti-G4S linker mAbs and compared them with conventional detection reagents across multiple CAR T-cell products (**Figures 1A–C**). Manufactured CAR T-cell products prior to infusion were analyzed with representative gating for CAR^+^ T-cell detection shown in **Figure 1A**. In the CD19-directed product axi-cel, staining with anti-Whitlow/218 produced CAR^+^ frequencies equivalent to anti-CD19 Ag and anti-idiotype CD19.FMC63 mAb. Similarly, anti-G4S mAb reliably identified CAR^+^ cells at frequencies matching anti-CD19 Ag and the anti-idiotype CD19.FMC63 mAb for tisa-cel. Comparable results were observed in the BCMA-directed product ide-cel, where anti-Whitlow/218 staining detection frequencies closely matched anti-BCMA Ag. This was consistent with dual-epitope CAR T-cell product cilta-cel, where the percentages of detected CAR^+^ T cells were comparable using anti-G4S and anti-BCMA Ag. Notably, in BCMA-directed products, anti-F(ab′)_2_ demonstrated lower CAR^+^ frequencies, pointing to lower sensitivity compared to both Ag and linker-specific reagents (**Figure 1B**). Moreover, we evaluated anti-G4S linker mAb in an investigational CLDN6-directed CAR T-cell product to assess the detection performance of linker-specific mAbs in investigational CAR T-cell constructs. Staining with anti-G4S linker mAb identified CAR^+^ T cells at frequencies closely matching anti-F(ab′)_2_ staining (**Figure 1C**). Collectively, these data demonstrate that anti-Whitlow/218 and anti-G4S mAbs enable reliable, antigen-independent detection of CAR T cells across various CAR constructs.

### Detection specificity and sensitivity are comparable between anti-linker and anti-idiotype reagents

**Figure 2:**
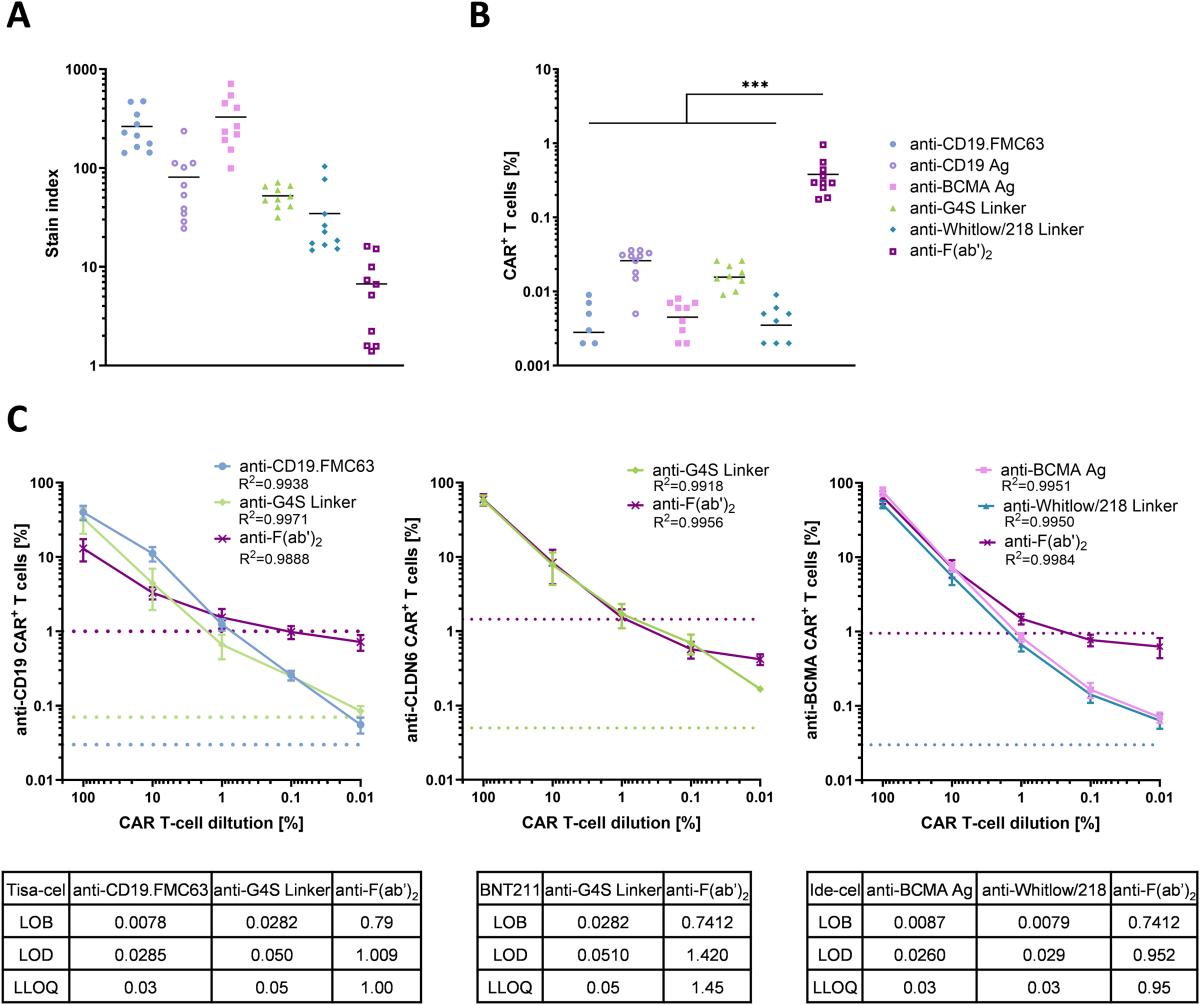
Specificity and sensitivity of CAR detection reagents. **(A)** The graph depicts the stain index for each CAR detection reagent from PBMCs (n=10 patient samples). The stain index is calculated as the difference between mean fluorescence intensity (MFI) of CAR^+^ and CAR^-^ T-cells, divided by twice the standard deviation (SD) of the CAR^-^ population. Black bars indicate the mean, each symbol represents a single patient. **(B)** Background staining as CAR^+^ events from healthy donor (HD) PBMCs (n=10) who have never received CAR T-cell therapy for the respective CAR detection reagent. Black bars indicate the mean of the background signal, each symbol represents one donor. ***P < 0.001 (One-way ANOVA, paired, Tukey). **(C)** CAR T-cell products were serially spiked into healthy donor (HD) PBMCs to generate mixtures ranging from 100% to 0.01% CAR T-cell dilution (undiluted CAR T-cell products were defined as 100%). Samples were plotted on a logarithmic scale to determine the lower limit of quantification (LLOQ) for each monoclonal antibody (mAb). The LLOQs (dotted lines) were defined as the last dilution above the limit of detection (LOD) for each detection reagent with a coefficient of variation of 30–35%. Each symbol represents the mean ± SD (n=3) measurements for each detection reagent and correlating cell dilution. Tables indicate corresponding limit of blank (LOB), LOD and LLOQ values for each detection reagent for the CAR T-cell products tisa-cel, BNT211 and ide-cel.

To assess analytical performance, we compared the specificity and sensitivity of linker-specific mAbs with established Ag reagents. Staining intensity can be quantified by the stain index (SI), defined as the ratio of antibody signal relative to background fluorescence (37, 40). Among the tested reagents, anti-BCMA Ag and anti-idiotype CD19.FMC63 mAb exhibited the highest mean SIs (3547.3 and 263.3, respectively), followed by anti-CD19 Ag (80.8), anti-G4S (52.1), and the anti-Whitlow/218 (34.6). The anti-F(ab′)_2_ antibody displayed the lowest SI (6.7), indicating poor discrimination between positive and negative populations (**Figure 2A**). Background reactivity was quantified by the LOB, defined as the highest apparent signal in true negative samples (41). The most favorable LOB values (<1 x 10^-4^) were obtained for anti-Whitlow/218 mAb, anti-idiotype CD19.FMC63 mAb, and anti-BCMA Ag, indicating minimal nonspecific staining. Slightly higher LOBs were observed for anti-G4S (2.8 x 10^-4^) and anti-CD19 Ag (4.2 x 10^-4^), while anti-F(ab′)_2_ exhibited a markedly higher LOB (7.4 x 10^-3^) (**Figure 2B**). Reagent sensitivity was evaluated by determining the LLOQ, defined as the lowest antibody concentration that can be quantified with the precision defined by a coefficient of variation of 30-35%. The LLOQ was analyzed in serial dilutions of CAR T-cell products and determined based on the dilution step above the limit of detection (LOD) (38, 39). Independent of target antigen, linker-specific mAbs achieved high sensitivity, with comparable LLOQs of anti-G4S and anti-Whitlow/218, respectively, to antigen-specific reagents including anti-idiotype CD19.FMC63 mAb or anti-BCMA Ag. In contrast, anti-F(ab′)_2_ consistently exhibited substantially higher LLOQs across all CAR products (**Figure 2C**). Together, these findings demonstrate that linker-specific mAbs achieve detection specificity and sensitivity equivalent to established Ag reagents, with the advantage of being target independent.

### Anti-linker mAbs perform well across CAR constructs in real-time monitoring

**Figure 3:**
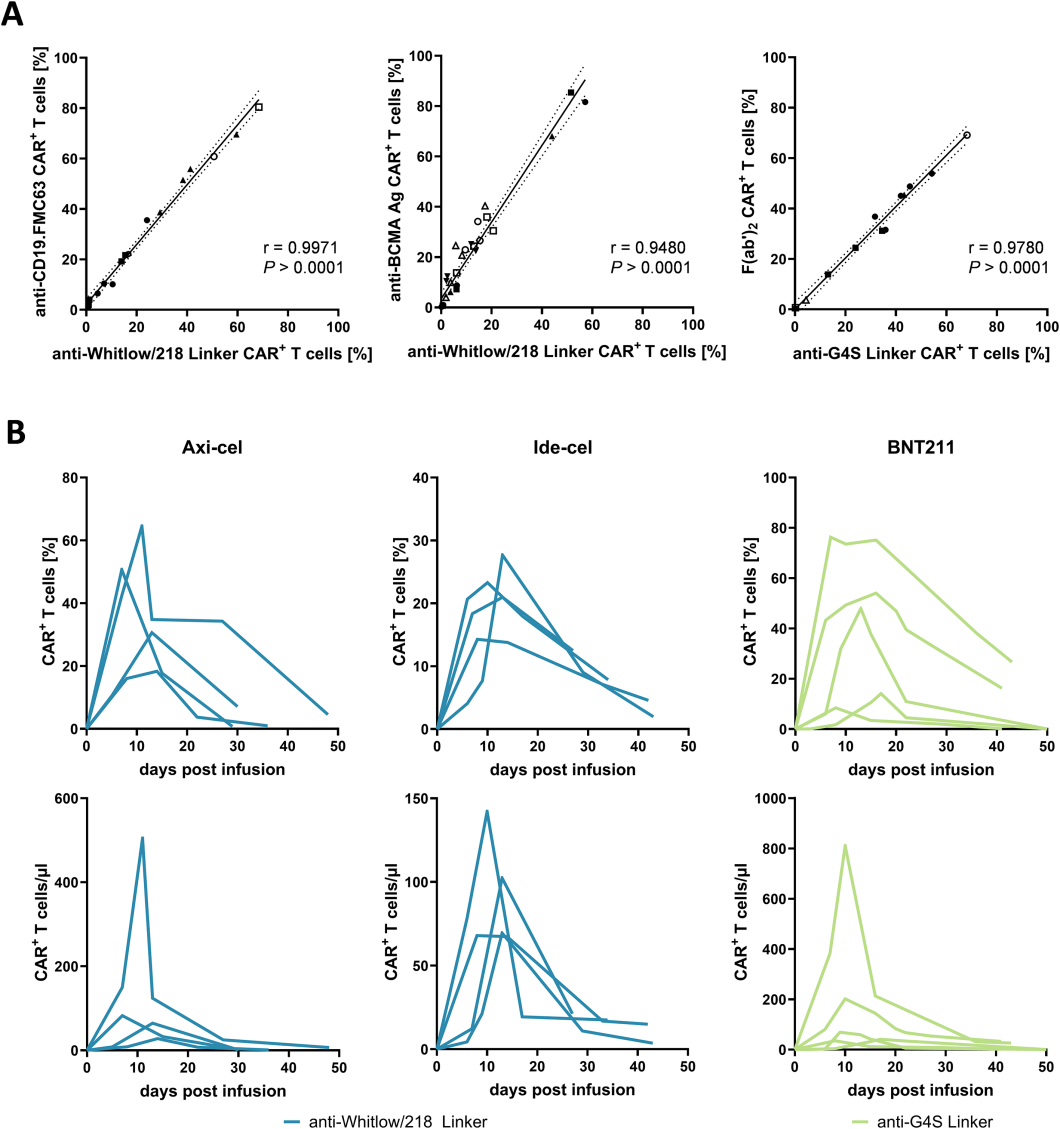
CAR T-cell monitoring in real-world patients using linker-specific mAbs. **(A)** Correlation of the rate of CAR^+^ T cells using linker-specific mAbs versus CAR-specific reagents. The percentage of CAR^+^ T cells detected with linker-specific mAbs (x-axis) is plotted against those detected with conventional CAR mAbs (y-axis). Correlation analyses are shown for three independent patient cohorts (left: n=5; middle: n=7; right: n=7 distinct patients, each measured over time). Patients do not overlap between panels. Each symbol represents one patient within a panel, with 5 longitudinal samples per patient plotted. Symbols across the three panels do not indicate the same individual patient. Correlations were assessed using Spearman’s rank correlation coefficient. **(B)** Longitudinal monitoring of CAR T-cell kinetics in patients post CAR T-cell therapy by linker-specific detection. PBMCs from patients with lymphoma (n = 4), multiple myeloma (n = 4), or solid tumors (n = 5) were collected at defined time points after infusion of the respective CAR T-cell products (axi-cel, ide-cel, and CLDN6-CAR T, respectively). Graphs show the percentage (top) and absolute number (bottom) of CAR T cells detected using anti-Whitlow/218 (blue) or anti-G4S (green) linker mAbs.

To further assess the comparability of linker-specific mAbs and established CAR detection reagents, we performed correlation analyses of CAR T-cell frequencies measured by linker mAbs and conventional reagents in a real-world monitoring setting over time. Notably, the percentage of CAR^+^ T-cells detected with anti-G4S and anti-Whitlow/218 linker mAbs correlated well with those obtained using respective conventional reagents, demonstrating that linker-specific mAbs detect CAR^+^ populations as effectively as established CAR detection reagents (**Figure 3A**). As multiple measurements per patient were included, observations were not independent and results should therefore be interpreted as exploratory. Reliable CAR detection reagents are essential for longitudinal patient monitoring to consistently quantify CAR T-cell expansion, subsequent persistence, and gradual decline over time. To assess key performance parameters, we analyzed kinetics of CAR T-cell percentages (upper row) and absolute CAR T-cell numbers (lower row) in patients over a period of 35–55 days post-infusion. In lymphoma patients (n=4) treated with the CD19-directed CAR T-cell product axi-cel, and in MM patients (n=4) treated with the BCMA-directed CAR product ide-cel, staining with the anti-Whitlow/218 linker mAb revealed patient-specific expansion patterns, including the characteristic peak and subsequent decline. Both closely matched longitudinal monitoring with CAR detection reagents anti-idiotype CD19.FMC63 and anti-BCMA Ag, respectively, confirming the consistency of linker-based detection in a longitudinal clinical setting (**Figure 3B** left, middle panel, **Supplementary Figure 1** left, middle panel). To explore whether this approach is also applicable to CAR constructs beyond hematologic targets, we extended the analysis to CAR T cells directed against the solid tumor antigen CLDN6. Measured real-time in five patients, the anti-G4S linker mAb enabled robust detection of CAR T cells, aligning with the expected expansion pattern (**Figures 3B**, right panel). This was consistent with the measured CAR T-cell kinetics with the anti-F(ab′)_2_ antibody (**Supplementary Figure 1**, right panel). Taken together, these results prove that linker-specific mAbs not only correlate with conventional CAR detection reagents at single time points but also reliably quantify CAR T cells over time and across different antigen targets, supporting their suitability for standardized longitudinal monitoring in clinical studies.

### Anti-linker mAbs enable target-independent CAR T-cell comparison using high-dimensional flow cytometry

**Figure 4:**
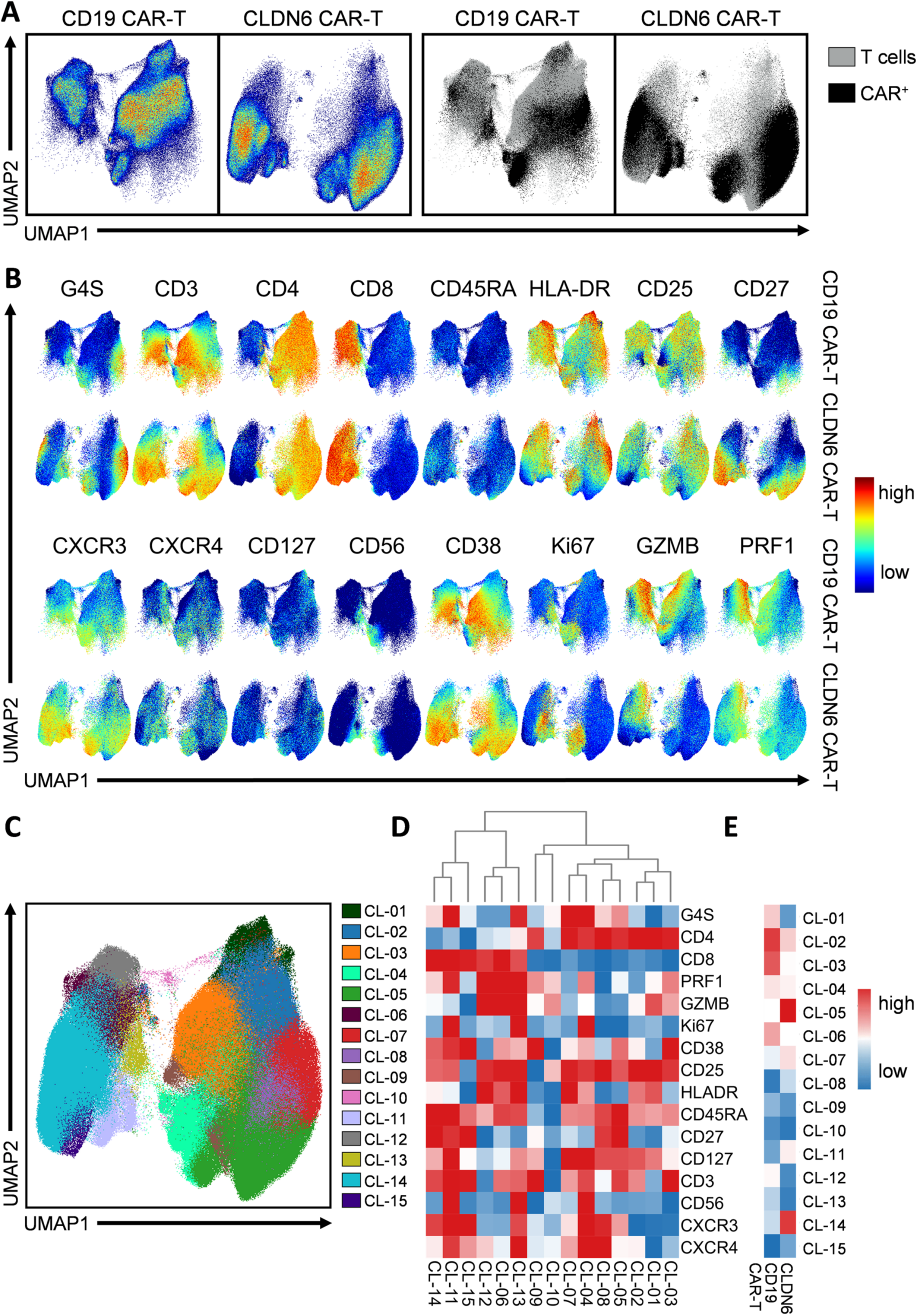
Performance of Linker-specific mAbs in high-dimensional flow cytometry. CD19 CAR-T and CLDN6 CAR-T cell products were stained with a viability dye, the anti-G4S Linker mAb, and the indicated antibodies. Viable CD3^+^ T cells (see **Figure 1** for gating) were used for an unsupervised clustering analysis. **(A)** Uniform Manifold Approximation and Projection (UMAP) plots show CD19 CAR-T (left) and CLDN6 CAR-T cell (right) products (n = 4 each). Left panels display cell density–based UMAP representations of all CD3^+^ T cells, while right panels depict CAR expression overlaid on the same UMAPs, with CAR^+^ T cells shown in black and non-CAR T cells in grey. **(B)** Expression maps of selected phenotypic and functional markers overlaid onto the UMAP space for CD19- or CLDN6-targeting CAR T cells, respectively. **(C)** UMAP-overlay including unsupervised clustering combining both, CD19 CAR-T and CLDN6 CAR-T cell products. **(D)** Clustered heatmap illustrating the expression profiles of key markers across meta-clusters (CL01–CL15). **(E)** Heatmap of the relative abundance of each cluster within CD19 CAR-T and CLDN6 CAR-T cell products.

To further validate their utility, we developed a 17-color high-dimensional flow cytometry panel to analyze the applicability of linker-specific mAbs in CD19- and CLDN6-directed CAR T cells. UMAP visualization revealed distinct phenotypic regions corresponding to CD19 CAR-T or to CLDN6 CAR-T cells, respectively (**Figure 4A**). Importantly, both CAR constructs were specifically and reliably detected with the anti-G4S linker mAb, with partially overlapping expression signals for CD19 CAR-T and CLDN6 CAR-T cells in the UMAP projection. Data were concatenated from four patients per construct. To visualize inter-patient variability, **Supplementary Figure 2** presents the same UMAP projections with patient-specific color overlays, demonstrating that each dataset contributed comparably to the overall clustering structure. Interestingly, key phenotypic and functional markers exhibited similar expression patterns between CD19 CAR-T and CLDN6 CAR-T cells except for CD27 expression, which was higher in CLDN6 CAR-T cells and GZMB expression levels, which were higher in CD19 CAR-T cells (**Figure 4B**). Dimensional reduction defined 15 distinct clusters in the combined population of both, CD19 CAR-T and CLDN6 CAR-T cells (**Figure 4C**). Within the joint dataset, all 15 clusters were defined by distinct individual maker signatures generally well conserved throughout the two distinct products (**Figure 4D**). However, marked differences between the two products were highlighted and predominantly found in clusters 2, 3, 5, and 14(**Figure 4E**). The granular identification of product-spanning as well as product-specific clusters confirm that linker-specific mAbs enable robust, antigen-independent detection of CAR T cells, while preserving the ability to resolve phenotypic and functional heterogeneity within high-dimensional flow cytometry datasets.

### Anti-linker mAbs provide versatile CAR detection in immunofluorescence and across species

**Figure 5:**
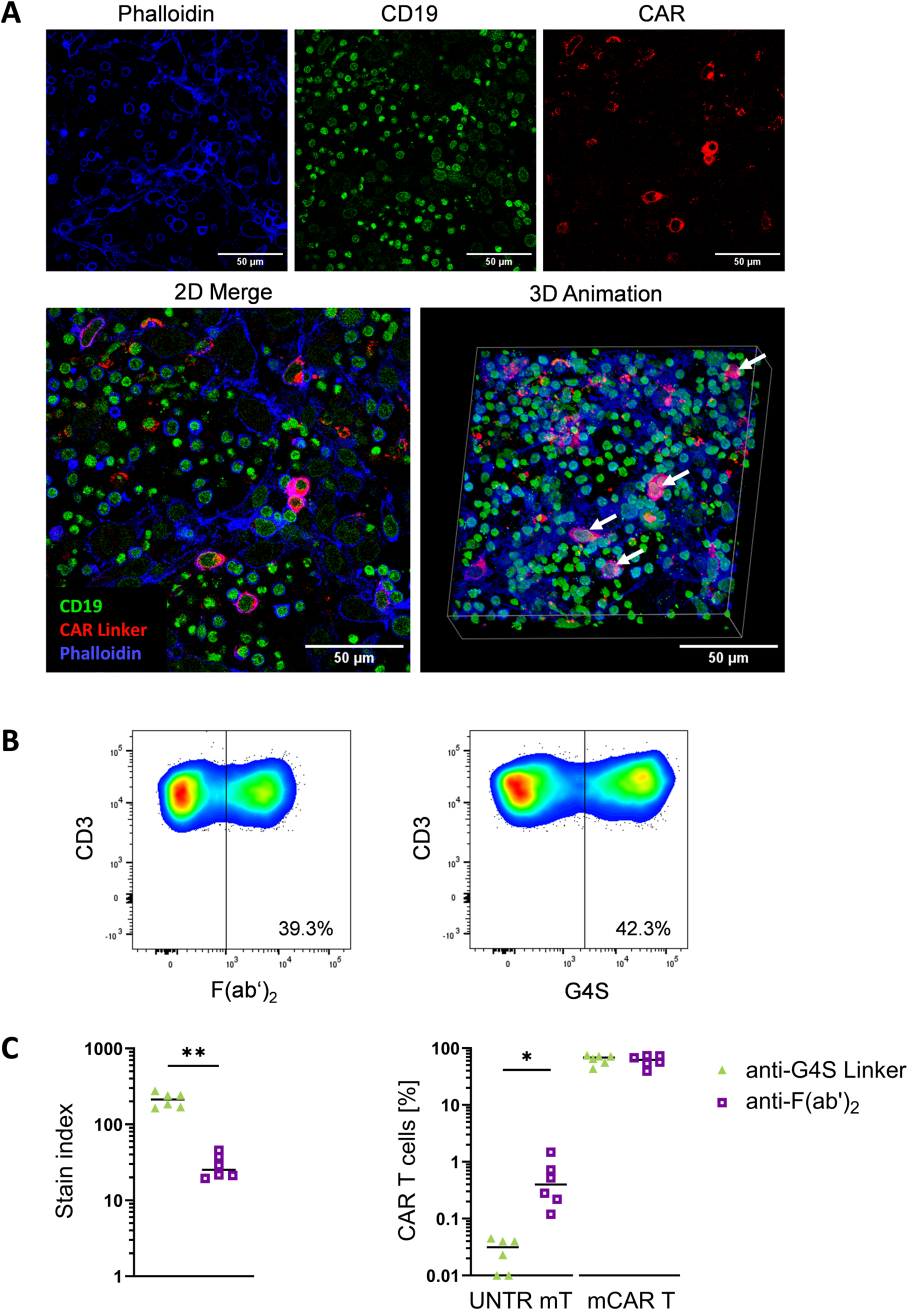
Versatility of linker mAbs across experimental models. **(A)** Confocal microscopy of a 3D co-culture model comprising bone marrow-derived stromal cells (BMSCs) (Phalloidin, blue), chronic lymphocytic leukemia (CLL) B cells (CD19^+^, green) and anti-CD19 CAR T cells stained with the anti-Whitlow/218 linker (red). Co-localizing CLL and anti-CD19 CAR T cells are indicated by white arrows in the animated 3D-image. **(B)** Representative flow cytometry density plots depict staining of murine CAR^+^ T-cells using the anti-F(ab′)_2_ (left) or the anti-G4S Linker mAb (right). **(C)** The stain index was calculated for the anti-G4S linker and anti-F(ab′)_2_ mAb in murine CAR^+^ T-cells (left). Percentage of CAR^+^ T cells of all murine T cells and the background signal of untransduced (UNTR) murine T cells (right) for the anti-G4S linker and anti-F(ab′)_2_ antibody, respectively, are shown. Each dot represents a biological replicate of one mouse (n=6). P < 0.05 (*), P < 0.01 (**) (Welch‘s t-test).

**Figure 6:**
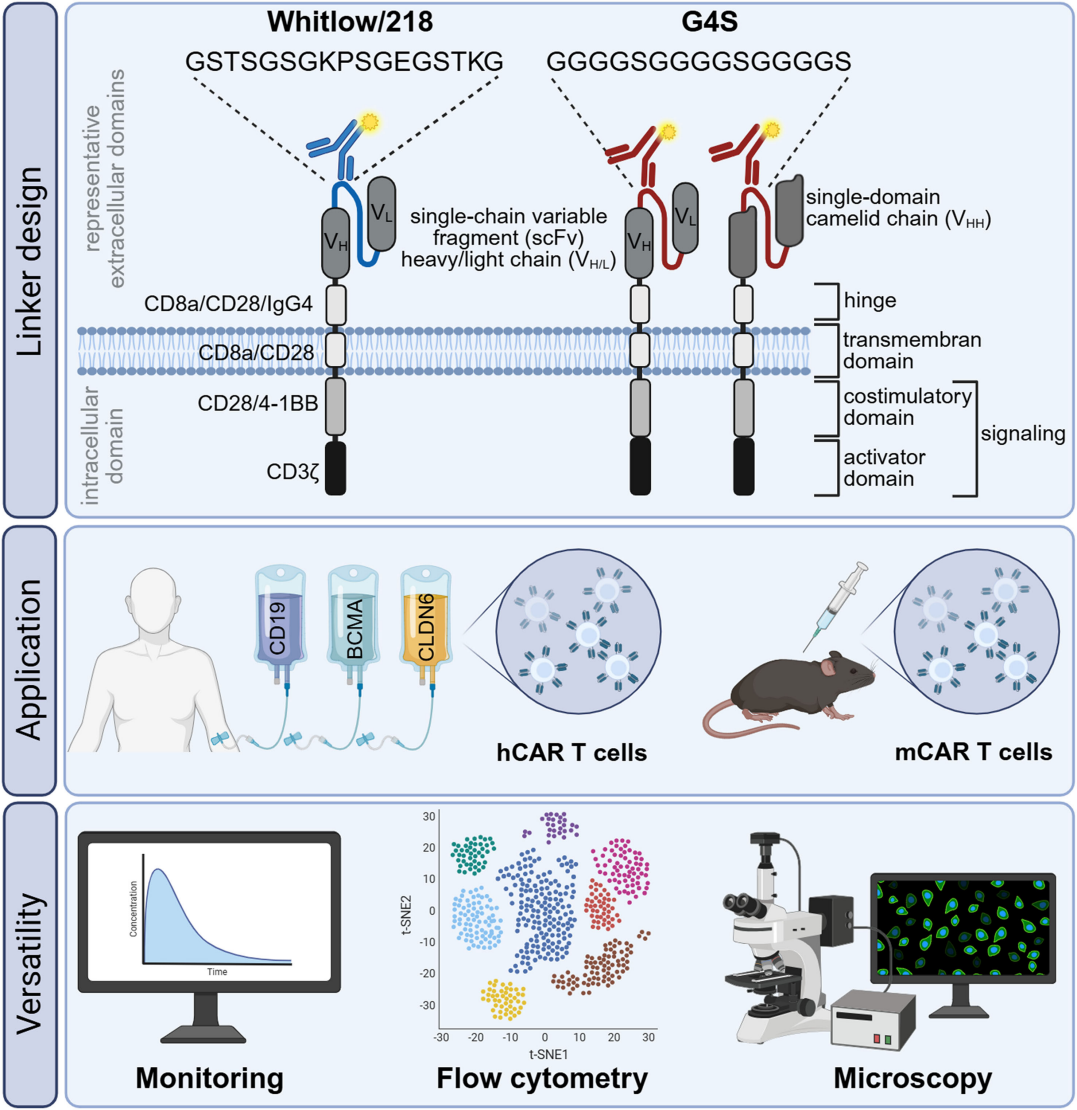
Visual abstract. Overview of CAR T-cell constructs, the application and the versatility of linker mAbs. CAR constructs incorporate different antigen-binding domains (scFv or VHH) connected by Whitlow/218 or G4S linkers and include hinge–transmembrane regions with CD28/4-1BB and CD3ζ signaling domains (upper panel). These CAR designs are applied to generate human and murine CAR T cells targeting antigens such as CD19, BCMA, and CLDN6 (middle panel). Functional characterization using linker m-Abs includes *in vitro* and *in vivo* applications and multiparametric analyses using clinical monitoring assays, high-dimensional flow cytometry, and microscopy (lower panel).

To extend the application of linker-specific mAbs beyond cell suspension-based measurements, we performed immunofluorescence staining of *in vitro* generated co-cultures of HD-derived anti-CD19 CAR T cells, patient-derived CLL cells, and the stromal cell line HS-5. Representative immunofluorescence images demonstrate robust and specific detection of anti-CD19 CAR T cells using anti-Whitlow/218 mAb (**Figure 5A**). Co-staining with phalloidin allowed visualization of HS-5 stromal cells and overall cell morphology, while CD19 staining specifically identified the malignant B-cells. Importantly, merged 2D images and 3D reconstruction of z-stacks allowed differentiation of cells in overlapping layers, revealed close spatial proximity of CAR T cells with tumor cells suggesting specific target cell recognition by the Whitlow/218 linker mAb (**Figures 5A**, merge). Lastly, we determined cross-species use of linker-specific mAbs in an anti-murine CD19 CAR construct (**Figures 5B, C**). As in anti-human CARs, we calculated the SI for anti-G4S linker mAb and anti-F(ab′)_2_ antibody to determine the signal intensity of the stained cell population (37, 40). Consistent with our results in patient-derived CAR T cells (**Figure 2A**) the anti-G4S linker mAb exhibited a significant higher SI compared to the anti-F(ab′)_2_ antibody, thus, demonstrating enhanced ability to distinguish CAR^+^ T cells from background also in a fully murine system (**Figures 5C**, left). Analysis of LOB in murine untransduced (UNTR) T cells revealed a lower background staining for anti-G4S linker mAb, while positive staining of freshly manufactured murine anti-CD19 CAR T cells was comparable for both mAbs (**Figures 5C**, right). These results demonstrate that linker-specific mAbs are a versatile, reliable tool for CAR T cell detection, applicable not only to human but also to murine CAR T cells, supporting their wide applicability in both preclinical and clinical research settings (**Figure 6**).

## Discussion

With the rapid growth of CAR T-cell research and clinical studies, the need for sensitive, versatile, and reproducible detection methods of CAR T cells in the patient blood keeps increasing. Reliable CAR monitoring is crucial for evaluating product quality, accurate *in vivo* tracking, providing direct insights when comparing clinical response with treatment related toxicities, and lastly, enabling precision downstream analyses (21, 42). Linker-specific mAbs targeting conserved sequences within CAR constructs offer a promising universal approach to CAR T-cell detection. In our study, we evaluated two linker-specific mAbs detecting either the Whitlow/218 linker or the G4S linker, across multiple CAR constructs, target antigens species, and test modalities, and compared their performance with established commercial reagents.

Our results show that linker-specific mAbs provide robust detection for both CD19 and BCMA targeting products, yielding comparable CAR^+^ frequencies to commonly used reagents targeting the CAR binding domain. While anti-idiotype mAbs are highly product-specific as they only recognize a single scFv and are therefore limited in the detection to a single target moiety such as FMC63 (26). The anti-Whitlow/218 and anti-G4S mAbs bind the conserved linker domain in CAR constructs, thus providing antigen-independent detection even in products that are based on camelid antibodies such as Cilta-cel. Furthermore, the simple detection method facilitates standardization of cell-monitoring across constructs, clinical studies, or study sites (12, 22, 33). In contrast, the polyclonal anti-F(ab′)_2_ antibody demonstrated lower detection sensitivity compared to linker-specific mAbs and anti-BCMA Ag. This observation is consistent with previous findings reporting limited accuracy and reproducibility of anti- F(ab′)_2_ compared to linker-specific mAbs (22). Given the rapid expansion of novel CAR T-cell therapies targeting diverse antigens, generated from various species, we further assessed the applicability of linker-specific mAbs for the investigational product BNT211, and found a highly sensitive and specific detection of CLDN6-targeting CAR T cells. This finding supports the utility of linker-specific mAbs for monitoring CAR T cells in patients with solid tumors and highlights their value as ready-to-use tools for novel CAR constructs and preclinical and clinical studies, where anti-idiotype mAbs or recombinant protein-based reagents are frequently not available (22, 26). Importantly, as development of new CAR antigens is rapidly progressing, linker-specific mAbs can overcome the inherent challenge of generating matched purified recombinant proteins or conjugates for detection, offering a universal and adaptable approach to CAR T-cell monitoring that is independent of antigen specificity.

Assessment of SI, LOB and LLOQ is critical for evaluating sensitivity and specificity, particularly in CAR T cell persistence when they are present at low frequencies (37, 40). Slightly lower SI values observed with linker mAbs could reflect steric hindrance of accessibility of the linker domain, which may limit antibody binding efficiency. Although the linker sequence is present, its structural orientation or the location in between heavy and light chain with possibly limited surface exposure could reduce effective antibody recognition, resulting in lower detection signals despite comparable CAR expression levels (43). In contrast, relatively high background levels were previously reported for direct CAR detection reagents (24). While linker-targeting mAbs have demonstrated high specificity for detecting various scFv-based CARs, comprehensive data on their background staining characteristics remain limited (12, 22). A recent study reported off-target staining of non-transduced PBMCs using various CAR detection reagents, including anti-linker mAbs, although no statistical analysis was provided (33). This underscores the importance of empirically determining the LOB for each detection reagent. In our study, both linker-specific mAbs demonstrated favorable low LOB values (<0.05%), comparable to those of Ag reagents, indicating that anti-linker mAbs can achieve high specificity for CAR detection without generating false-positive signals from background noise. Over time, CAR T-cell numbers typically decline *in vivo* with long-term persistence in peripheral blood often occurring at very low frequencies, requiring reliable monitoring with highly sensitive detection reagents (44–46). By determining the LLOQ, we demonstrated that linker-specific mAbs exhibit detection sensitivity comparable to both anti-CD19 Ag and anti-idiotype CD19.FMC63 mAb. This sensitivity enables accurate detection of CAR T cells even at low frequencies, supporting the suitability of anti-linker mAbs for clinical monitoring. Furthermore, the strong correlation observed between linker-specific and commonly used CAR-detection reagents highlights the reliability and versatility of linker-based detection. Notably, this correlation was consistent across CAR constructs targeting CD19, BCMA, or CLDN6, indicating that linker-specific mAbs can provide reliable, sensitive, and specific antigen-independent quantification of CAR T cells and suggesting their powerful interchangeability for CAR T-cell detection. Such universality is particularly valuable for longitudinal clinical monitoring, where consistent and reproducible detection over time is essential for assessing CAR T-cell expansion, persistence and functional dynamics (12, 22, 42). Furthermore, the comparable performance of linker-specific mAbs and established reagents supports their implementation in both preclinical studies and patient monitoring especially when no Ag-reagent is available, providing a standardized approach that could reduce variability between assays and across study centers. Taken together, these findings highlight the potential of linker-based detection not only as a research tool but also as a practical option for routine clinical immune monitoring, facilitating precise assessment of real-time CAR T-cell kinetics and supporting the development of personalized adoptive immunotherapies.

A major advance in flow cytometry-based CAR T-cell analysis over qPCR- or ddPCR-based monitoring analysis is the ability to determine the cellular phenotypic and functional properties of CAR T cells. By using high-dimensional flow cytometry, we demonstrated that the anti-linker mAb is compatible with multi-marker panels, enabling consistent CAR identification and resolution of 17 distinct marker expressions within different CAR^+^ T-cell products. This also permitted identification of discrete clusters, delineation of marker expression across clusters, and quantitative comparison of cluster abundances between CD19- and CLDN6-targeting CAR T-cell products. The ability to dissect phenotypic subpopulations and link these to differences in the antigen target, manufacturing, or anticipated *in vivo* behavior, is essential for future studies. High-dimensional immunophenotyping provides refined product characterization, monitoring CAR T cells post-infusion, and ultimately correlating phenotypic features with clinical parameters (42). As the field advances toward a deeper mechanistic understanding and increasingly personalized adoptive cell therapies, embedding CAR detection into comprehensive immune-profiling platforms will be crucial for connecting manufacturing, biology, and clinical response.

Extending this concept beyond flow cytometry, we evaluated the utility of anti-linker mAbs in imaging-based applications. Immunofluorescence staining with the anti-Whitlow/218 mAb demonstrated CAR expression and colocalization of CAR T cells with malignant B cells, indicating spatial association compatible with potential cell–cell interactions. Anti-linker mAbs enable immunofluorescence-based visualization of CAR T-cell localization and interactions in complex culture systems, extending linker-based CAR detection beyond flow cytometry to support assessment of CAR T-cell distribution and dynamics in preclinical models and potentially patient-derived samples. To date, detection of murine CAR T cells has primarily been demonstrated using the F(ab′)_2_ antibody (31). By directly comparing anti-F(ab′)_2_ with the anti-linker mAb in murine CAR T cells, we observed even higher detection efficiency and reduced background staining with the anti-linker mAb. This establishes linker-specific mAbs as a true cross-platform detection tool, enabling direct comparison between preclinical murine CAR T-cell models and human clinical CAR T-cell products using the same standardized detection system. Linker-specific mAbs further support harmonization of CAR T-cell research by enabling applications such as magnetic enrichment of CAR T cells using the anti-G4S linker mAbs, preserving viability and function and making them suitable for downstream single-cell omics analyses (47). Similarly, linker-based reagents have been applied in ELISA and Western blot assays, as well as in immunoprecipitation of CAR molecules from cell lysates, which can be valuable for proteomic studies or assessing CAR stability (12). A limitation using linker mAbs to detect commercial CAR T-cell products is that linker sequences are often disclosed in patents and not readily accessible, necessitating empirical testing to identify suitable linker-specific mAbs. Additionally, CAR constructs that lack conventional amino acid linkers or incorporate novel linker sequences pose further challenges for this approach. Moreover, it should be noted, that current evidence suggests that linker-specific mAbs may not detect CAR-expressing cells in FFPE tissue, likely due to epitope masking from formaldehyde crosslinking that hides the linker sequence (33).

Despite these limitations, linker-specific mAbs demonstrate robust, sensitive, and antigen-independent detection of CAR T cells across multiple constructs, species, and methods. Their compatibility with high-dimensional flow cytometry, imaging, and downstream assays highlights their versatility as a cross-platform tool. Overall, the anti-Whitlow/218 and anti-G4S mAbs provide a standardized and reliable approach for both preclinical and clinical CAR T-cell monitoring and facilitate consistent longitudinal CAR T-cell detection in the clinical and the preclinical setting.

## Data Availability

The original contributions presented in the study are included in the article/**Supplementary Material**. Further inquiries can be directed to the corresponding author.
