# Supplementary material for: Universal high-sensitivity CAR T-cell monitoring by targeting linker sequences
Source: Front Immunol. 2026 Mar 25;17:1787951. doi: 10.3389/fimmu.2026.1787951 (PMC13057459; doi:10.3389/fimmu.2026.1787951)
Supplement: Supplementary file 1 [file Presentation1.pptx]

## Slide 1
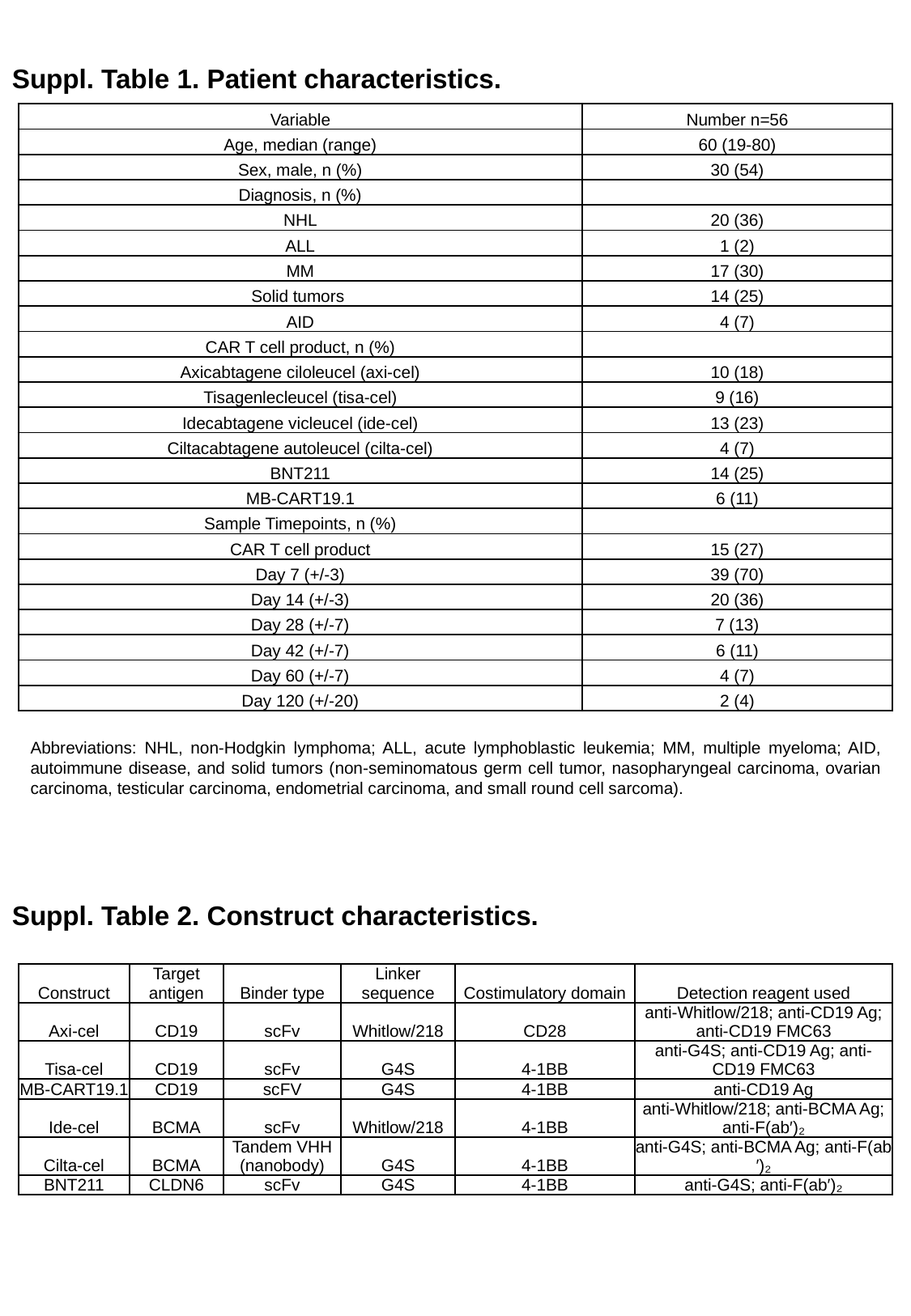

Suppl. Table 1. Patient characteristics.
| Variable | Number n=56 |
| --- | --- |
| Age, median (range) | 60 (19-80) |
| Sex, male, n (%) | 30 (54) |
| Diagnosis, n (%) | |
| NHL | 20 (36) |
| ALL | 1 (2) |
| MM | 17 (30) |
| Solid tumors | 14 (25) |
| AID | 4 (7) |
| CAR T cell product, n (%) | |
| Axicabtagene ciloleucel (axi-cel) | 10 (18) |
| Tisagenlecleucel (tisa-cel) | 9 (16) |
| Idecabtagene vicleucel (ide-cel) | 13 (23) |
| Ciltacabtagene autoleucel (cilta-cel) | 4 (7) |
| BNT211 | 14 (25) |
| MB-CART19.1 | 6 (11) |
| Sample Timepoints, n (%) | |
| CAR T cell product | 15 (27) |
| Day 7 (+/-3) | 39 (70) |
| Day 14 (+/-3) | 20 (36) |
| Day 28 (+/-7) | 7 (13) |
| Day 42 (+/-7) | 6 (11) |
| Day 60 (+/-7) | 4 (7) |
| Day 120 (+/-20) | 2 (4) |
Abbreviations: NHL, non-Hodgkin lymphoma; ALL, acute lymphoblastic leukemia; MM, multiple myeloma; AID, autoimmune disease, and solid tumors (non-seminomatous germ cell tumor, nasopharyngeal carcinoma, ovarian carcinoma, testicular carcinoma, endometrial carcinoma, and small round cell sarcoma).
Suppl. Table 2. Construct characteristics.
| Construct | Target antigen | Binder type | Linker sequence | Costimulatory domain | Detection reagent used |
| --- | --- | --- | --- | --- | --- |
| Axi-cel | CD19 | scFv | Whitlow/218 | CD28 | anti-Whitlow/218; anti-CD19 Ag; anti-CD19 FMC63 |
| Tisa-cel | CD19 | scFv | G4S | 4-1BB | anti-G4S; anti-CD19 Ag; anti-CD19 FMC63 |
| MB-CART19.1 | CD19 | scFV | G4S | 4-1BB | anti-CD19 Ag |
| Ide-cel | BCMA | scFv | Whitlow/218 | 4-1BB | anti-Whitlow/218; anti-BCMA Ag; anti-F(ab′)₂ |
| Cilta-cel | BCMA | Tandem VHH (nanobody) | G4S | 4-1BB | anti-G4S; anti-BCMA Ag; anti-F(ab′)₂ |
| BNT211 | CLDN6 | scFv | G4S | 4-1BB | anti-G4S; anti-F(ab′)₂ |

## Slide 2
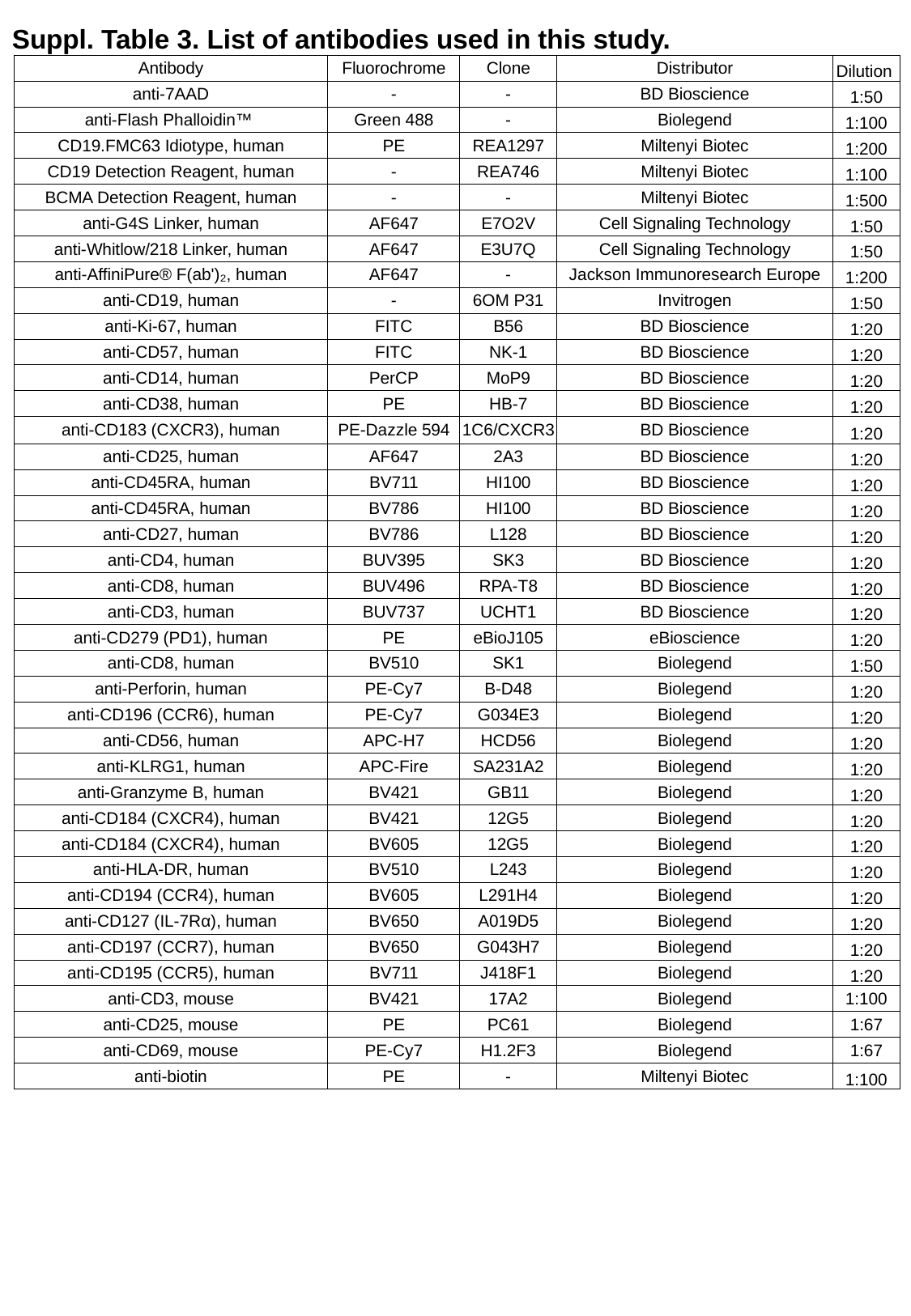

Suppl. Table 3. List of antibodies used in this study.
| Antibody | Fluorochrome | Clone | Distributor | Dilution |
| --- | --- | --- | --- | --- |
| anti-7AAD | - | - | BD Bioscience | 1:50 |
| anti-Flash Phalloidin™ | Green 488 | - | Biolegend | 1:100 |
| CD19.FMC63 Idiotype, human | PE | REA1297 | Miltenyi Biotec | 1:200 |
| CD19 Detection Reagent, human | - | REA746 | Miltenyi Biotec | 1:100 |
| BCMA Detection Reagent, human | - | - | Miltenyi Biotec | 1:500 |
| anti-G4S Linker, human | AF647 | E7O2V | Cell Signaling Technology | 1:50 |
| anti-Whitlow/218 Linker, human | AF647 | E3U7Q | Cell Signaling Technology | 1:50 |
| anti-AffiniPure® F(ab')₂, human | AF647 | - | Jackson Immunoresearch Europe | 1:200 |
| anti-CD19, human | - | 6OM P31 | Invitrogen | 1:50 |
| anti-Ki-67, human | FITC | B56 | BD Bioscience | 1:20 |
| anti-CD57, human | FITC | NK-1 | BD Bioscience | 1:20 |
| anti-CD14, human | PerCP | MoP9 | BD Bioscience | 1:20 |
| anti-CD38, human | PE | HB-7 | BD Bioscience | 1:20 |
| anti-CD183 (CXCR3), human | PE-Dazzle 594 | 1C6/CXCR3 | BD Bioscience | 1:20 |
| anti-CD25, human | AF647 | 2A3 | BD Bioscience | 1:20 |
| anti-CD45RA, human | BV711 | HI100 | BD Bioscience | 1:20 |
| anti-CD45RA, human | BV786 | HI100 | BD Bioscience | 1:20 |
| anti-CD27, human | BV786 | L128 | BD Bioscience | 1:20 |
| anti-CD4, human | BUV395 | SK3 | BD Bioscience | 1:20 |
| anti-CD8, human | BUV496 | RPA-T8 | BD Bioscience | 1:20 |
| anti-CD3, human | BUV737 | UCHT1 | BD Bioscience | 1:20 |
| anti-CD279 (PD1), human | PE | eBioJ105 | eBioscience | 1:20 |
| anti-CD8, human | BV510 | SK1 | Biolegend | 1:50 |
| anti-Perforin, human | PE-Cy7 | B-D48 | Biolegend | 1:20 |
| anti-CD196 (CCR6), human | PE-Cy7 | G034E3 | Biolegend | 1:20 |
| anti-CD56, human | APC-H7 | HCD56 | Biolegend | 1:20 |
| anti-KLRG1, human | APC-Fire | SA231A2 | Biolegend | 1:20 |
| anti-Granzyme B, human | BV421 | GB11 | Biolegend | 1:20 |
| anti-CD184 (CXCR4), human | BV421 | 12G5 | Biolegend | 1:20 |
| anti-CD184 (CXCR4), human | BV605 | 12G5 | Biolegend | 1:20 |
| anti-HLA-DR, human | BV510 | L243 | Biolegend | 1:20 |
| anti-CD194 (CCR4), human | BV605 | L291H4 | Biolegend | 1:20 |
| anti-CD127 (IL-7Rα), human | BV650 | A019D5 | Biolegend | 1:20 |
| anti-CD197 (CCR7), human | BV650 | G043H7 | Biolegend | 1:20 |
| anti-CD195 (CCR5), human | BV711 | J418F1 | Biolegend | 1:20 |
| anti-CD3, mouse | BV421 | 17A2 | Biolegend | 1:100 |
| anti-CD25, mouse | PE | PC61 | Biolegend | 1:67 |
| anti-CD69, mouse | PE-Cy7 | H1.2F3 | Biolegend | 1:67 |
| anti-biotin | PE | - | Miltenyi Biotec | 1:100 |

## Slide 3
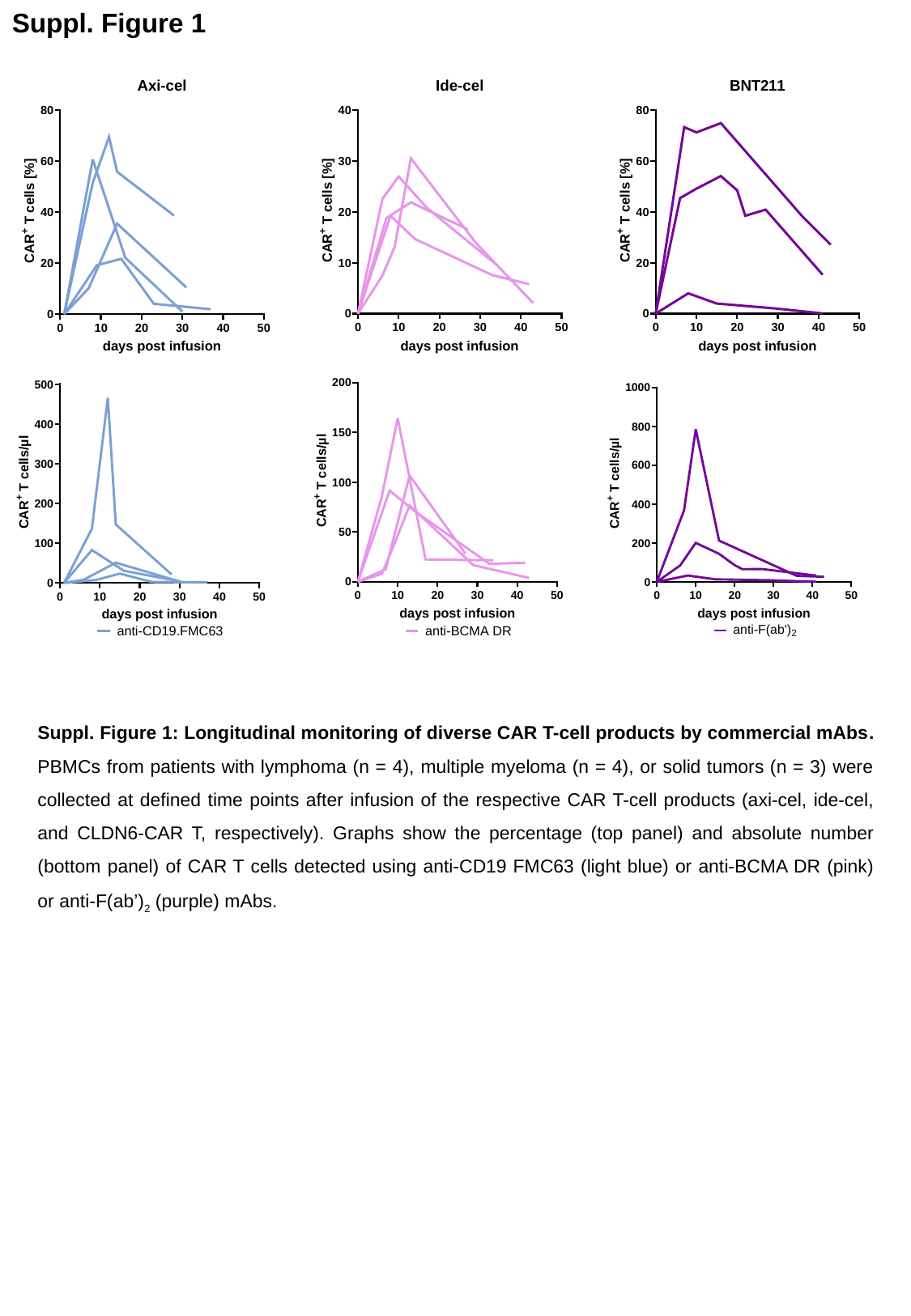

Suppl. Figure 1
Suppl. Figure 1: Longitudinal monitoring of diverse CAR T-cell products by commercial mAbs. PBMCs from patients with lymphoma (n = 4), multiple myeloma (n = 4), or solid tumors (n = 3) were collected at defined time points after infusion of the respective CAR T-cell products (axi-cel, ide-cel, and CLDN6-CAR T, respectively). Graphs show the percentage (top panel) and absolute number (bottom panel) of CAR T cells detected using anti-CD19 FMC63 (light blue) or anti-BCMA DR (pink) or anti-F(ab’)2 (purple) mAbs.

## Slide 4
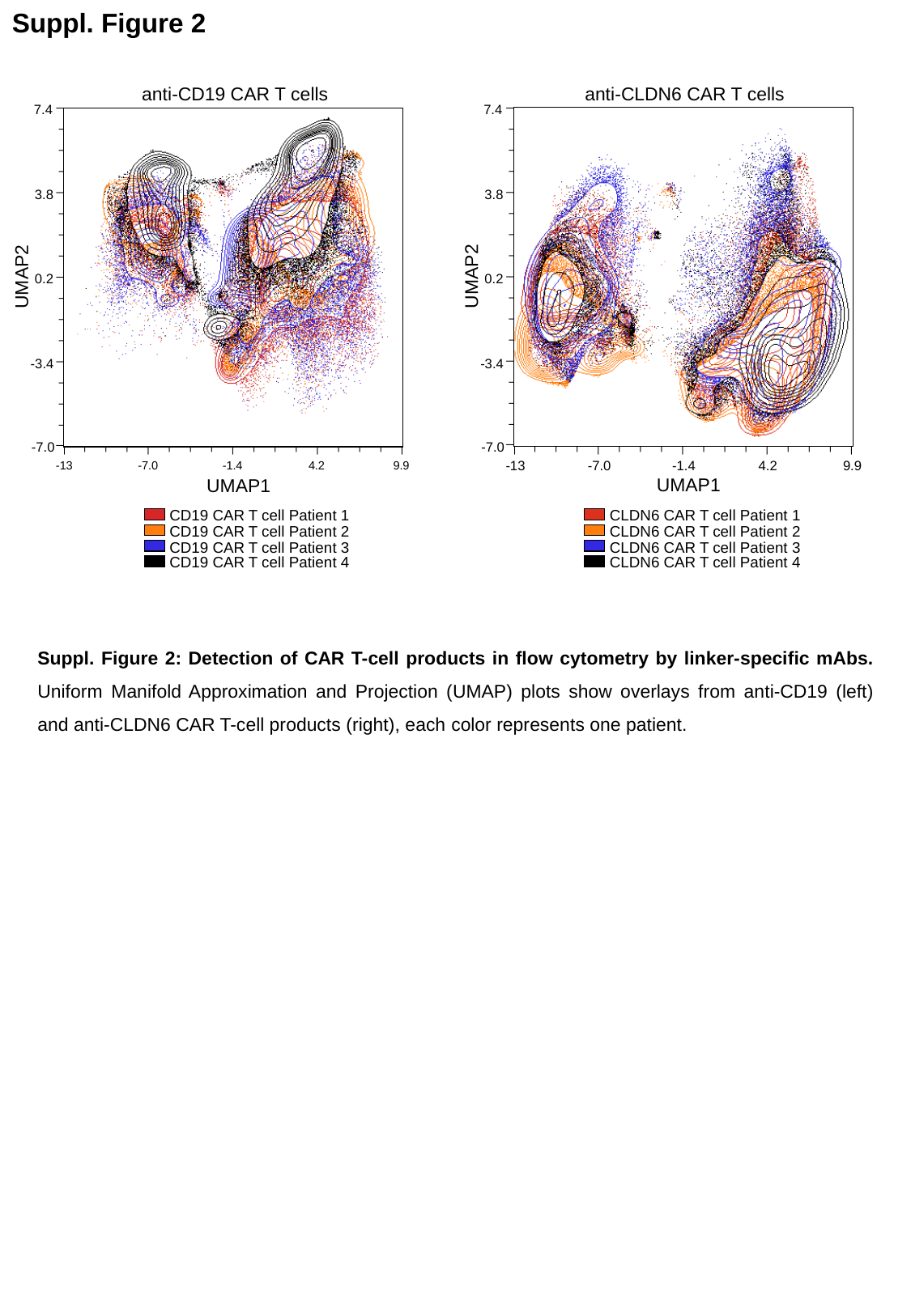

Suppl. Figure 2
anti-CD19 CAR T cells
anti-CLDN6 CAR T cells
7.4
3.8
0.2
-3.4
-7.0
7.4
3.8
0.2
-3.4
-7.0
UMAP2
UMAP2
9.9
-13
-7.0
4.2
-1.4
9.9
-13
-7.0
4.2
-1.4
UMAP1
UMAP1
CD19 CAR T cell Patient 1
CLDN6 CAR T cell Patient 1
CD19 CAR T cell Patient 2
CLDN6 CAR T cell Patient 2
CD19 CAR T cell Patient 3
CLDN6 CAR T cell Patient 3
CD19 CAR T cell Patient 4
CLDN6 CAR T cell Patient 4
Suppl. Figure 2: Detection of CAR T-cell products in flow cytometry by linker-specific mAbs. Uniform Manifold Approximation and Projection (UMAP) plots show overlays from anti-CD19 (left) and anti-CLDN6 CAR T-cell products (right), each color represents one patient.
